# Evaluation of voltage-dependent calcium channel γ gene families identified several novel potential susceptible genes to schizophrenia

**DOI:** 10.1038/srep24914

**Published:** 2016-04-22

**Authors:** Fanglin Guan, Tianxiao Zhang, Xinshe Liu, Wei Han, Huali Lin, Lu Li, Gang Chen, Tao Li

**Affiliations:** 1 Department of Forensic Psychiatry, School of Medicine & Forensics, Xi’an Jiaotong University, Xi’an, China; 2 Key Laboratory of National Ministry of Health for Forensic Sciences, School of Medicine & Forensics, Xi’an Jiaotong University, Xi’an, China; 3 Department of Psychiatry, School of Medicine, Washington University, Saint Louis, MO, USA; 4 Department of Forensic Medicine, School of Medicine & Forensics, Xi’an Jiaotong University, Xi’an, China; 5 Xi’an Mental Health Center, Xi’an, China

## Abstract

Voltage-gated L-type calcium channels (VLCC) are distributed widely throughout the brain. Among the genes involved in schizophrenia (SCZ), genes encoding VLCC subunits have attracted widespread attention. Among the four subunits comprising the VLCC (α − 1, α −2/δ, β, and γ), the γ subunit that comprises an eight-member protein family is the least well understood. In our study, to further investigate the risk susceptibility by the γ subunit gene family to SCZ, we conducted a large-scale association study in Han Chinese individuals. The SNP rs17645023 located in the intergenic region of *CACNG4* and *CACNG5* was identified to be significantly associated with SCZ (OR = 0.856, *P* = 5.43 × 10^−5^). Similar results were obtained in the meta-analysis with the current SCZ PGC data (OR = 0.8853). We also identified a two-SNP haplotype (rs10420331-rs11084307, P = 1.4 × 10^−6^) covering the intronic region of *CACNG8* to be significantly associated with SCZ. Epistasis analyses were conducted, and significant statistical interaction (OR = 0.622, *P* = 2.93 × 10^−6^, *P*_perm_ < 0.001) was observed between rs192808 (*CACNG6*) and rs2048137 (*CACNG5*). Our results indicate that *CACNG4*, *CACNG5*, *CACNG6* and *CACNG8* may contribute to the risk of SCZ. The statistical epistasis identified between *CACNG5* and *CACNG6* suggests that there may be an underlying biological interaction between the two genes.

Schizophrenia (SCZ) has a lifetime risk of approximately 1% and is a complex psychiatric disorder characterized by delusions, hallucinations, altered cognition, emotional reactivity and disorganized behavior^1^,^2^,^3^. Many epidemiological studies and recent genetic studies of association mapping have suggested that genetic and gene–environment interactions account for over 80% of the susceptibility of developing SCZ^4^,^5^. These studies have been accelerated by the widely used application of genome-wide association studies (GWASs) in SCZ^6^,^7^,^8^,^9^,^10^. To date, the Psychiatric Genomics Consortium (PGC) has published three mega-analyses on SCZ^11^,^12^,^13^. A recent multi-stage GWAS of SCZ by PGC demonstrated that 108 schizophrenia-associated loci met genome-wide significance in a sample of 36,989 cases and 113,075 controls, 83 of which have not been previously reported^13^. However, current replicable GWAS results account for only a small percentage of the estimated heritability^14^, and their systematic biological interpretation is lacking. Focusing on a specific GWAS-based variant might be misleading because the results of a GWAS contains many false positives^15^,^16^ and genetic heterogeneity of SCZ exists in different populations^17^. Thus, follow-up studies are essential to confirm the GWAS findings and extend them.

Voltage-gated L-type calcium channels (VLCC) are distributed widely throughout the brain and are critical for mediating intracellular Ca^2 + ^influx in response to action potentials at the synapse. VLCCs have an important role in N-methyl-d-aspartate (NMDA) receptor-independent synaptic plasticity processes^18^. Among the genes involved in SCZ, genes encoding VLCC subunits have attracted widespread attention. Recent GWASs have indicated that the *CACNA1C* gene, encoding an alpha-1 subunit of VLCC, is a susceptibility gene for SCZ in individuals of European ancestry^11^,^12^,^13^. Our previous study has confirmed the association of *CACNA1C* with SCZ in a Han Chinese population^19^, and some neuroimaging studies have also documented the effects of *CACNA1C* variants on a range of structural and functional brain phenotypes, including the circuitry involved in emotion processing, executive function, attention and memory^20^,^21^,^22^. However, among the four subunits comprising the mammalian VLCC (alpha-1, alpha-2/delta, beta and gamma), the gamma subunit is the least well understood. The calcium channel gamma subunits comprise an eight-member protein family that shares a common topology consisting of four transmembrane domains and intracellular N- and C-termini. Some phylogenetic, bioinformatic and functional studies have indicated that they are a functionally diverse protein family and that *CACNG1-CACNG8* are all co-expressed in adult brains as an important mechanism for regulating Ca^2+^ channel function^23^,^24^. *CACNG2* has been reported to be a susceptibility locus for SCZ in some populations^25^,^26^. However, given that some studies suggest an involvement of calcium-dependent regulatory processes in prefrontal-hippocampal network plasticity^27^,^28^ and the pathophysiology of SCZ^20^,^21^, it is still necessary to explore the potential association between common variants in the *CACNG* genes and SCZ. In the present study, to further investigate the risk of susceptibility to SCZ due to *CACNG1-CACNG8*, we conducted a large-sample association analysis in Han Chinese individuals.

## Materials and Methods

### Subjects

A total of 10,039 individuals, including 7521 healthy controls (3,903 females and 3,618 males; range of age 18–51 years; mean age 37.8 ± 9.6) and 2518 patients with SCZ (1,307 females and 1,211 males; range of age 18–51 years; mean age 36.9 ± 9.3), were recruited for this study. All patients were recruited from the inpatient and outpatient clinical services of a psychiatric unit at the Xi’an Mental Health Center and were diagnosed by at least two experienced psychiatrists based on the Diagnostic and Statistical Manual of Mental Disorders (DSM-IV) criteria for SCZ. All unrelated healthy controls were selected from a combination of local volunteers and blood transfusion donors; those with a personal family history of mental illness in the previous three generations and with current or past evidence of psychoses were ruled out from the present study. All subjects enrolled from the city of Xi’an in Shaanxi Province were of Han descent, and we excluded anyone not born locally or whose immediate family members from the previous three generations were not born locally. This research was performed in accordance with the ethical guidelines of the Declaration of Helsinki (version 2002) and was approved by the Medical Ethics Committee of Xi’an Jiaotong University. All participants completed written informed consent forms.

### Genotyping

Because *CACNG1*, *CACNG4* and *CACNG5* are located next to each other on chromosome 17, we searched for all SNPs in the region covering the three genes with minor allele frequencies (MAF) > 0.05 and heterozygosity (>0.2) in the HapMap Han Chinese in Beijing (CHB) population (Release 28). SNPs with pair-wise tagging and *r*^2^ ≥ 0.5 were used as the criteria when selecting tag SNPs in the LD blocks. The same criteria were used to select tag SNPs within *CACNG6*-*8* on chromosome 19, *CACNG2* on chromosome 22 and *CACNG3* on chromosome 16. Given that the lengths of *CACNG2* and *CACNG3* are both more than 100 kb, we only selected the tag SNPs (tagging number > 3) in the LD blocks. Based on above criteria, a custom set of 35 tag SNPs were finally selected in our study, and the distributions of these SNPs is shown in Supplementary Table S1. DNA was extracted from whole blood according to the standard protocol of the DNA Isolation Kit for Mammalian Blood (Tiangen Biotech Co., Ltd, Beijing, China). The DNA was stored at −80°C for genotyping. The genotyping was conducted for all of the SNPs using the Sequenom MassARRAY matrix-assisted laser desorption ionization-time of flight mass spectrometry platform (Sequenom, San Diego, CA, USA) on the genomic DNA isolated from the peripheral leukocytes. The final data were released using Typer Analyzer software. The reliability of the subsequent statistical analysis was ensured, as the final genotype call rate of each SNP was greater than 99.3% and the overall genotyping call rate was 99.5%. Additionally, 5% of random samples were repeated, and the results were 100% concordant.

### Statistical analysis

#### Power analysis and quality control

We conducted a comprehensive power analysis using Genetic Power Calculator^29^ based on our sample size and genotyped markers to estimate the statistical power. The results are summarized in Supplemental Table S2. As shown in the table, for our sample size (10,039), we were able to achieve a statistical power greater than 80% to detect a common risk genetic variant that had an effect range from 1.2 to 1.3. Genetic markers that had an MAF greater than 0.05 and that passed the Hardy-Weinberg equilibrium test were included for further analysis.

#### Association Analysis

We performed both single-marker-based and haplotype-based analyses to detect the potential association signals between our genotyped SNPs and the SCZ disorder status. We conducted single-marker-based analysis by fitting a logistic model and the age and sex of the individuals were included in the model as two covariates to account for potential confounding effects. The SNPs were coded in three different models: additive, dominant and recessive. In the additive model, the individual’s genotype is coded as 0, 1 and 2 when there are 0, 1 or 2 minor alleles. In the dominant model, the individual’s genotype is coded as 0 when it is a homozygote of a major allele and 1 when it is a heterozygote or homozygote of a minor allele. In the recessive model, the individual’s genotype is coded as 1 when it is a homozygote of a minor allele and 0 otherwise. The LD blocks were constructed using the default algorithm taken from Gabriel *et al.*^30^ We generated 95% confidence bounds on D’, and each comparison was called “strong LD” when the confidence bounds had an upper bound ≥ 0.98 and a lower bound ≥ 0.7. A block was created if 95% of the informative comparisons were “strong LD”. Plink^31^ was utilized for logistic model fitting, linkage disequilibrium (LD) blocks construction and haplotype-based association. A Bonferroni correction was performed to address the potential multiple comparison problems.

#### Epistasis Analysis

We conducted two-way epistasis analysis to detect the potential gene-by-gene interactions among the eight *CACNG* genes. Logistic models were fitted as shown in the following functions,


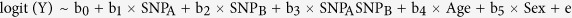


where Y is the SCZ disorder status. The test for interaction is based on the coefficient b_3_. Two covariates, sex and age, were added in the logistic model similar to the single-marker-based analysis. The SNP pairs were tested using the logistic model in an exhaustive way. Although SNP pairs located on the same chromosome were removed during analysis, this method still produces a large number of statistical tests (432 tests in total). To reduce the inflated type I errors generated by multiple comparison, we performed 1,000 permutations by shuffling the case-control labels of our samples, and the null distribution was created by using the most significant *P* values in each permutation cycle. The corrected *P* values for each SNP pair were calculated using this newly created null distribution to account for potential problems of multiple comparison. We utilized statistical computing software R^32^ to conduct this epistasis analysis.

#### Imputation and Meta-Analysis

We conducted imputation for a 1 Mb region around SNP rs17645023. We utilized genetic software IMPUTE2^33^ for the imputation and SNPTEST^34^ for the follow-up dosage data association test. The CHD + CHB HapMap3 dataset served as the reference data. The info metric was used as a method to remove poorly imputed SNPs from our association results. There is no universal cutoff value for post-imputation SNP filtering so we assessed different info thresholds by checking whether they produced sensible Q-Q plots (Supplemental Figure S1). We chose 0.4 as our info metric threshold by the Q-Q plots. In addition to the imputation analysis, we also conducted Meta-analysis for SNP rs17645023 using five GWAS datasets from the PGC. The R package meta^35^ was utilized for summarizing and virtualization of the meta-analysis.

#### Bioinformatics analysis

We examined the targeted SNPs and/or genes using several bioinformatics tools and databases. We utilized the protein-protein interaction database STRING (http://string-db.org/) to explore the potential interactions of our targeted genes. The Regulome DB (http://regulomedb.org/) was used to predict the potential functional consequences of the identified risk SNPs. This database is a web-based bioinformatics tool integrated with multiple types of data (including ChIP-seq, DNase-seq, and eQTLs) from the Encyclopedia of DNA Elements (ENCODE) project^36^.

## Results

### Association analysis

All of our genotyped SNPs passed our quality control criteria (Supplemental Table S3). The *P* value threshold for single-marker-based analysis was 0.001 (≈0.05/35). The SNP rs17645023 located on the intergenic region of *CACNG5* and *CACNG4* was identified to be significant in both the additive and dominant models (Table 1). The genotype AA (or allele A) was significantly overrepresented in SCZ individuals. We also identified a significant protective effect of minor allele T in both the additive (OR = 0.86, *P* = 5.43 × 10^−5^) and dominant (OR = 0.85, *P* = 3.46 × 10^−4^) models. The full results of single marker based association analysis are summarized in Supplemental Table S4. Four LD blocks were constructed using our dataset. In the haplotype-based analysis, we found a two-SNP haplotype (rs10420331-rs11084307, *P* = 1.4 × 10^−6^) to be significantly associated with the SCZ disease status. This SNP pair covers the intronic region of gene *CACNG8*. The full results of haplotype analysis are summarized in Table 2.

### Epistasis analysis

**Figure 1:**
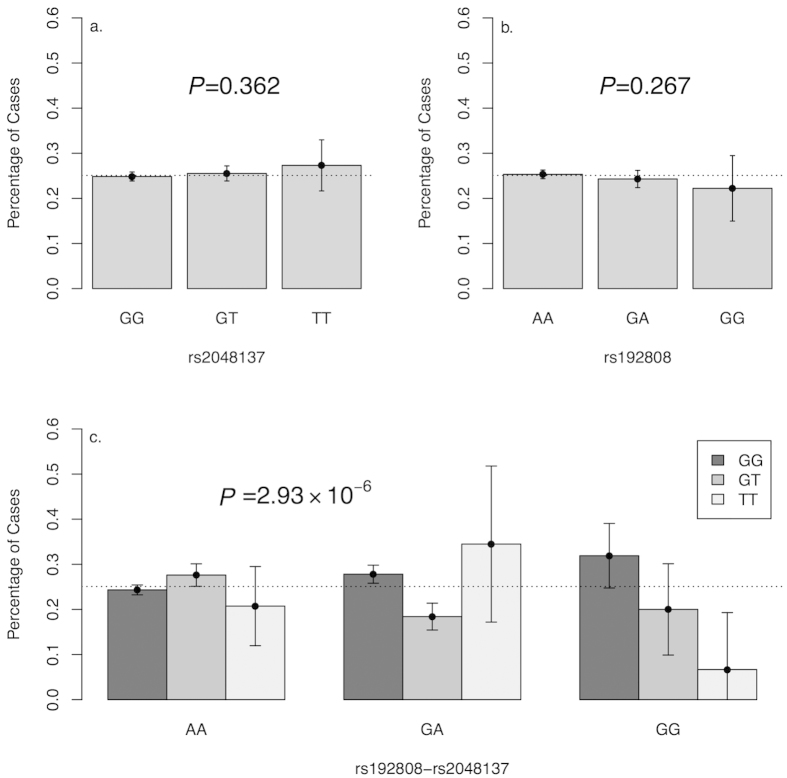
Epistasis analysis results for rs192808 and rs2048137. (**a**) Average percentage of SCZ patients for different genotypes of SNP rs2048137. (**b**) Average percentage of SCZ patients for different genotypes of SNP rs192808. c. Average percentage of SCZ patients for the combination genotype of rs192808 and rs2048137. The average percentage of SCZ patients in all samples is indicated with the dotted line. The significant *P* values based on the additive model are shown in each plot.

The two-way epistasis analysis included 432 SNP pairs, and the full results are summarized in Supplemental Table S5. We identified significant statistical interaction (OR = 0.622, *P* = 2.93 × 10^−6^, *P*_perm_ < 0.001) between rs192808 (*CACNG6*) and rs2048137 (*CACNG5*) (Fig. 1). Both double homozygote GG-TT and double heterozygote GA-GT (rs192808-rs2048137) were overrepresented in control samples.

### Imputation and Meta-Analysis

**Figure 2:**
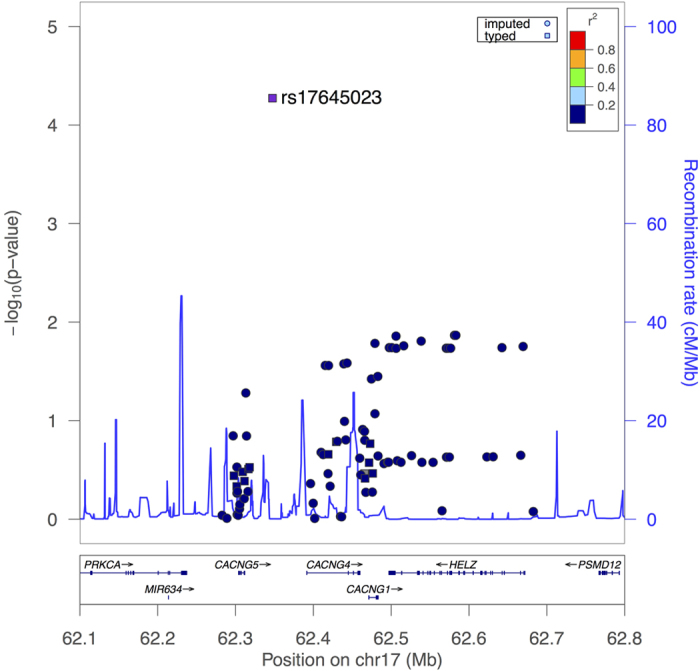
Regional association plot constructed based on the 1 Mb region around SNP rs17645023. The genotyped SNPs are indicated as squares and the imputed SNPs are indicated as circles. The most significant genotyped SNP was chosen as a reference SNP in plots (rs17645023).

**Figure 3:**
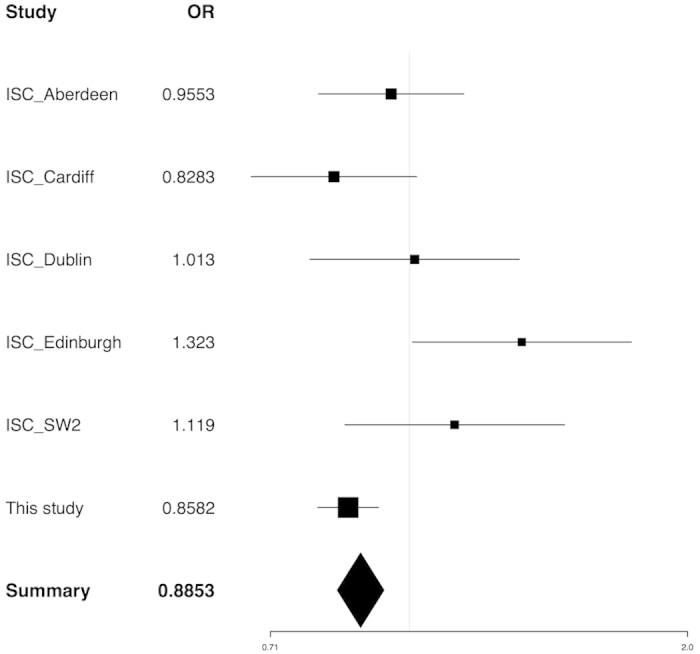
The forest plot of the meta-analysis for SNP rs17645023 using five PGC GWAS datasets.

A total of 73 imputed SNPs passed our quality control criteria (info > 0.4). The full results of imputation association are summarized in Supplemental Table S6. No new significant SNPs were identified with imputation analysis. The regional association plot based on both imputed and genotyped SNPs is shown in Fig. 2. The results of the meta-analysis are presented in Fig. 3, and the summarized result of the meta-analysis focused on rs17645023 was still significant with OR = 0.8853. The detailed information on the datasets used in the meta-analysis is summarized in Supplemental Table S7.

### Bioinformatics Analysis

**Figure 4:**
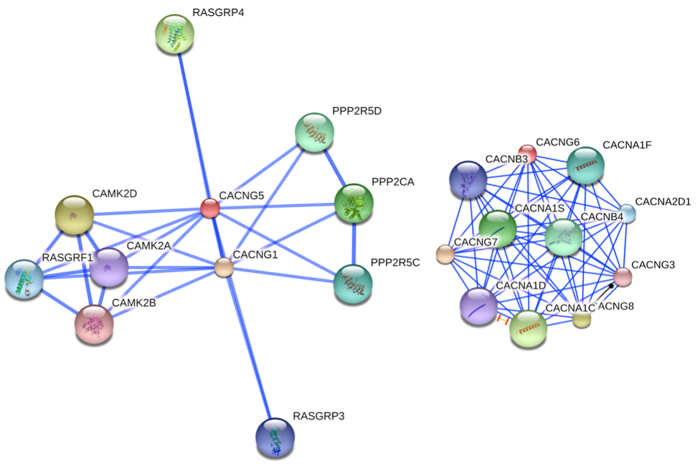
(**A**) The protein-protein interactions of CACNG5. (**B**) The protein-protein interactions of CACNG6.

We explored the protein-protein interaction for the two genes with significant statistical epistasis evidence (*CACNG5* and *CACNG6*) (Fig. 4). No direct evidence was found to support the protein-protein interaction between the two genes. The most related calcium channel voltage-dependent gamma subunits for *CACNG6* are *CACNG7* and *CACNG8*. Conversely, for *CACNG5*, the most related protein is encoded by *CACNG1*. We investigated the potential functional consequences of rs17645023 in Regulome DB. This SNP has a Regulome DB score of 4, which means the potential biological function of this SNP is supported by both transcription factor (TF) binding and DNase peak data. The ChIP-seq data indicates that this SNP is located within the protein binding region of the transcription factor CEBPB in all three cell types (A549, HeLa-S3 and IMR90). In addition, rs17645023 is also located within the DNase hypersensitive region in all eight cell types (T47d, Sknmc, A549, Hvmf, Hpde6e6e7, Fibrop, Fibropag20443 and Fibrobl).

## Discussion

One major finding in our study is the identification of a significant association between an intergenic SNP, rs17645023, and SCZ disease status. The potential association of this SNP with SCZ was first reported by Curtis *et al.* in 2011 using 523 SCZ cases and 505 controls with European ancestry (*P* < 10^−6^)^37^. The T allele at SNP rs17645023 is protective in both our study and the study of Curtis *et al.* However, the follow-up GWAS with the largest sample sizes has not replicated this result^13^. Our study is the first conducted with individuals of Chinese Han ancestry to replicate the significant association of rs17645023 and SCZ. The finding in this study of the association between rs17645023 and SCZ has generated more questions than answers. As an intergenic SNP, it is difficult to map to a specific gene. In the study conducted by Curtis *et al.*, rs17645023 was originally mapped to *CACNG5,* which is located 35 kb upstream from rs17645023. However, rs17645023 was also shown to be located approximately 44 kb upstream of *CACNG4*. It should not be assumed that this SNP is mapped to *CACNG5* simply because it is located 9 kb closer than *CACNG4*. Fortunately, we now have more efficient bioinformatics tools and large-scale datasets (such as ENCODE). A bioinformatics analyses indicates that rs17645023 may affect the binding of the transcription factor CEBPB, suggesting that it is more reasonable to map this SNP to *CACNG4,* which is located downstream of the SNP.

Our haplotype-based analyses provided some clues to a potential association between *CACNG8* and SCZ. To our knowledge, our study is the first to identify such a link. Although more studies are still needed to illustrate the specific effects of the *CACNG8* gene on SCZ, a general discussion based on previous literature might be helpful to provide some clues. Rouach *et al.* studied the role of the Cacng8 protein in mice^38^. The authors found that mice deficient in the Cacng8 protein showed a differential regulation of functional α-amino-3-hydroxy-5-methyl-4-isoxazolepropionic acid receptor (AMPA receptor) in the extrasynaptic and synaptic pools. These findings indicate that the Cacng8 protein may act as a critical protein for AMPA receptor expression and distribution in the hippocampus of mice. Interestingly, multiple studies have demonstrated that SCZ is related to brain region- and subunit-specific abnormalities in the expression of the AMPA receptor subunits^39^,^40^. In this sense, the *CACNG8* gene may affect the onset and development of SCZ through its effect on the expression and distribution of AMPA receptors.

Numerous studies have indicated that the epistasis effects can account for a large portion of the genetic effects for a complex disorder^41^,^42^,^43^. Early studies have provided statistical evidence for the epistasis between *DISC1*, *CIT* and *NDEL1* and risk for SCZ^44^. Our two-way interaction analyses identified a significant SNP pair (rs192808- rs2048137, *CACNG6*-*CACNG5*). Despite the problems associated with multiple comparisons, the result of the permutation analysis (*P*_perm_ < 0.001) indicates that this signal cannot be attributed to chance. Nevertheless, the identification of a statistical epistasis among susceptible genes does not illustrate their underlying biological interactions. As discussed above, *CACNG5* was reported to be associated with SCZ in the study of Curtis *et al.*^37^, although it is unlikely the SNP rs17645023 maps to *CACNG5*. *CACNG6* was previously reported to be associated with aspirin-intolerance in asthmatics in a Korean population^45^, and to date, no evidence has shown a potential link between *CACNG6* and SCZ. Based on sequence homology and chromosomal linkage, the calcium channel γ subunits can be divided into three clusters: (γ_1_,γ_6_), (γ_5_,γ_7_) and (γ_2_,γ_3_,γ_4_,γ_8_)^24^. Therefore, the proteins encoded by *CACNG5* and *CACNG6* belong to different clusters of calcium channel γ subunits. Our bioinformatics analysis using STRING found no direct evidence for the protein-protein interaction between the two different proteins encoded by *CACNG5* and *CACNG6*. More studies are still needed to clarify the underlying biological interactions between the two proteins.

A major strength of our study is the candidate gene-based study design. Although a GWAS has a much larger genomic coverage, its results are susceptible to many type I errors. Additionally, applying the Bonferroni correction for the inflated type I errors will result in the loss of several signals with moderate effects. Our candidate gene-based design avoids this disadvantage by focusing on a couple of functionally related genes and controlling the number of genotyped markers to a reasonable level. Another strength of our study is that we have a much larger sample size (~10,000) in comparison to other similar studies. A power analysis showed that this sample size enables us to detect moderate genetic effects with ORs as small as 1.15. There are also several limitations of our study. The coverage of our genotyped SNPs is a major disadvantage of this study. In the present study, we genotyped only 35 SNPs (~4 SNPs per gene) within the calcium channel γ subunit gene family (*CACNG1*-*CACNG8*). Therefore, it is doubtful that our study is complete because potential significant signals may be missed due to the low coverage of our genetic markers. Although this limitation can be partly compensated through imputation analyses, our results should be confirmed in other cohorts and populations using high-density marker panels and large-scale genotyping to provide more confident evidence for the association of the SNPs with SCZ. In addition to the low coverage of genetic markers, the population stratification might be another limitation for our study. Unlike large-scale genetic studies, which enable researchers using high-density marker data to apply some statistical intensity methods (e.g., genomic control, PCA) to correct the underlying population stratifications, we cannot perform any direct controls in our study because of the limited number of genotyped markers. Another limitation of this study is that in order to reduce the genotyping costs and maximize the genetic information, all SNPs genotyped in our study are tag SNPs in the LD blocks, and very few of them can be constructed into LD blocks again. As a result of this strategy, it is difficult to carry out haplotype-based analysis for the SNP of rs17645023 (due to the difficulties of constructing LD blocks), and we lost the chance to further validate our significant signal from a single-marker analysis in the haplotype-based analysis. In addition, although several quality control processes have been applied during the sample collection process to ensure the uniformity of the genetic background of our samples, this does not guarantee the complete removal of the confounding effects of different sub-populations especially considering the genetic diversity of the Han Chinese population. Therefore, our findings should be considered preliminary, and additional follow-up studies are required, including high-density mapping and targeted deep sequencing to provide additional information beyond studies focused on common variants and to undercover fundamental characteristics of any potential associations with SCZ. In addition, in addition to the calcium channel γ gene family, recent studies have also identified several susceptible genes for SCZ within the α and β gene families. Systematic studies focusing on all the calcium channel gene families are needed to illustrate the biomedical and the biological significance of these genes on SCZ.

In summary, our results indicated that *CACNG4*, *CACNG5*, *CACNG6* and *CACNG8* might be genes that contribute to the risk of SCZ. The statistical epistasis identified between *CACNG5* and *CACNG6* suggests that there is an underlying biological interaction between the two genes. However, more research is still needed to confirm our findings and to clarify the pathological mechanisms of the functional role of the calcium channel γ subunits gene family in SCZ and to eventually confer changes in clinical practice.

## Additional Information

**How to cite this article**: Guan, F. *et al.* Evaluation of voltage-dependent calcium channel γ gene families identified several novel potential susceptible genes to schizophrenia. *Sci. Rep.* **6**, 24914; doi: 10.1038/srep24914 (2016).

## Supplementary Material

Supplementary Table S1-S7 & Figure S1

## Figures and Tables

**Table 1:** The results of a single-marker-based association analysis for rs17645023.

	Genotype (n = 10,039)			*P*_*allelic*_	*P*_additive_	*P*_dominant_	*P*_recessive_
	AA (%)	AT (%)	TT (%)				
SCZ (n = 2,518)	1,503 (59.7)	891 (35.4)	124 (4.9)	**7.30 × 10**^**−5**^	**5.43 × 10**^**−5**^	**3.46 × 10**^**−4**^	0.004
Controls (n = 7,521)	4,189 (55.7)	2,843 (37.8)	489 (6.5)				

The significant results are highlighted in bold.

**Table 2:** The results of a haplotype-based analysis.

SNPS	HAPLOTYPE	F_A	F_U	CHISQ	*P*
rs2048137-rs740805	OMNIBUS	–	–	1.607	0.448
	GA	0.156	0.151	0.576	0.448
	TA	0.268	0.276	1.388	0.239
	TG	0.577	0.573	0.261	0.609
rs11659136-rs2286678	OMNIBUS	–	–	0.992	0.609
	TG	0.259	0.253	0.587	0.444
	CG	0.192	0.189	0.155	0.694
	CA	0.550	0.558	0.965	0.326
rs11079671-rs4791016	OMNIBUS	–	–	1.303	0.521
	GA	0.146	0.142	0.425	0.514
	GC	0.083	0.079	0.725	0.395
	CC	0.771	0.779	1.218	0.270
rs10420331-rs11084307	OMNIBUS	–	–	26.960	**1.40 × 10**^**−6**^
	GT	0.376	0.386	1.548	0.213
	AT	0.058	0.041	26.830	**2.22 × 10**^**−7**^
	AC	0.566	0.574	0.906	0.341

The significant results are highlighted in bold.
