# Supplementary material for: Evaluation of voltage-dependent calcium channel γ gene families identified several novel potential susceptible genes to schizophrenia
Source: Sci Rep. 2016 Apr 22;6:24914. doi: 10.1038/srep24914 (PMC4840350; doi:10.1038/srep24914)
Supplement: Supplementary Table S1-S7 & Figure S1 [file srep24914-s1.pdf]

Title: Systematic evaluation of voltage-dependent calcium channel γ gene families based on GWAS identified several novel potential susceptible genes to schizophrenia

Author names and affiliations: Fanglin Guan [a,b,*], Tianxiao Zhang [c,*], Xinshe Liu [b,d], Wei Han [a,b], Huali Lin [e], Lu Li [b], Gang Chen [b] and Tao Li [b,d]

[a] Department of Forensic Psychiatry, School of Medicine & Forensics, Xi'an Jiaotong University, Xi'an, China;

[b] Key Laboratory of National Ministry of Health for Forensic Sciences, School of Medicine & Forensics, Xi'an Jiaotong University, Xi'an, China;

[c] Department of Psychiatry, School of Medicine, Washington University, Saint Louis, MO, USA;

[d] Department of Forensic Medicine, School of Medicine & Forensics, Xi'an Jiaotong University, Xi'an, China;

[e] Xi'an Mental Health Center, Xi'an, China.

* These authors contributed equally to this work.

Corresponding Author:

Tao Li, M.D. & Ph.D., Department of Forensic Medicine, School of Medicine & Forensics, Xi'an Jiaotong University, Xi'an, Shaanxi, China, 710061.

Tel: 86-29-82655117; Fax: 86-29-82655472.

E-mail: litao050428@mail.xjtu.edu.cn

Supplemental Table S1. General information of the 35 SNPs genotyped in the study.

SNP	CHR	POS	Alleles	Func	Gene
rs11646957	16	24267309	A/C	untranslated-5	*CACNG3*
rs7204986	16	24312768	C/T	intron	*CACNG3*
rs4787985	16	24330095	C/T	intron	*CACNG3*
rs11074616	16	24340691	A/G	intron	*CACNG3*
rs2048137	17	64867456	G/T	intron	*CACNG5*
rs740805	17	64871070	C/T	intron	*CACNG5*
rs11659136	17	64878728	C/T	intron	*CACNG5*
rs2286678	17	64881075	A/G	intron	*CACNG5*
rs11079671	17	64886173	C/G	unknown	*CACNG5*
rs4791016	17	64887517	G/T	unknown	*CACNG5*
rs17645023	17	64917033	A/T	unknown	*CACNG5*
rs4790998	17	64988706	A/G	intron	*CACNG4*
rs8071936	17	64999003	G/T	intron	*CACNG4*
rs2885928	17	65035235	C/T	unknown	*CACNG1*
rs2159210	17	65035960	C/T	unknown	*CACNG1*
rs16960487	17	65041073	A/G	intron	*CACNG1*
rs3785579	17	65042501	C/G	intron	*CACNG1*
rs2363843	17	65045786	C/T	intron	*CACNG1*
rs11672982	19	54429198	G/T	intron	*CACNG7*
rs10411229	19	54434126	G/T	intron	*CACNG7*
rs11084305	19	54460053	G/T	unknown	*CACNG8*
rs10420331	19	54469068	A/G	intron	*CACNG8*
rs11084307	19	54481150	C/T	intron	*CACNG8*
rs192808	19	54510717	C/T	intron	*CACNG6*
rs459247	19	54515874	A/G	ncRNA,untranslated-3	*CACNG6*
rs140034	22	36955367	C/T	unknown	*CACNG2*
rs2235682	22	36983428	A/G	intron	*CACNG2*
rs2283986	22	36989406	A/G	intron	*CACNG2*
rs4821513	22	37013819	C/T	intron	*CACNG2*
rs2283992	22	37015271	A/T	intron	*CACNG2*
rs5756268	22	37020141	C/T	intron	*CACNG2*
rs12628674	22	37063016	C/T	intron	*CACNG2*
rs738516	22	37087493	A/C	intron	*CACNG2*
rs2092662	22	37088339	G/T	intron	*CACNG2*
rs2267361	22	37100260	A/G	near-gene-5	*CACNG2*

Supplemental Table S2. Results of the systematic power analysis. Statistical powers greater than 80% are highlighted in bold.

OR \ MAF	0.05	0.1	0.15	0.2	0.25	0.3	0.35	0.4	0.45
1.05	0.06	0.07	0.10	0.14	0.18	0.16	0.13	0.11	0.09
1.1	0.08	0.16	0.28	0.42	0.57	0.50	0.38	0.28	0.21
1.15	0.11	0.30	0.55	0.76	**0.90**	**0.84**	0.70	0.55	0.40
1.2	0.17	0.49	0.80	**0.95**	**0.99**	**0.97**	**0.91**	0.78	0.62
1.25	0.24	0.68	**0.94**	**0.99**	**1.00**	**1.00**	**0.98**	**0.92**	0.79
1.3	0.32	**0.83**	**0.99**	**1.00**	**1.00**	**1.00**	**1.00**	**0.98**	**0.90**
1.35	0.42	**0.93**	**1.00**	**1.00**	**1.00**	**1.00**	**1.00**	**1.00**	**0.96**
1.4	0.53	**0.97**	**1.00**	**1.00**	**1.00**	**1.00**	**1.00**	**1.00**	**0.99**
1.45	0.63	**0.99**	**1.00**	**1.00**	**1.00**	**1.00**	**1.00**	**1.00**	**1.00**
1.5	0.72	**1.00**	**1.00**	**1.00**	**1.00**	**1.00**	**1.00**	**1.00**	**1.00**

Supplemental Table S3. MAF and Hardy-weinberg equilibrium test results for the 35 genotyped SNPs.

CHR	SNP	POS	A1	A2	MAF	HWE
16	rs11646957	24267309	A	C	0.1942	0.6289
16	rs7204986	24312768	C	T	0.1570	0.6663
16	rs4787985	24330095	T	C	0.2342	0.9742
16	rs11074616	24340691	A	G	0.1876	0.8804
22	rs140034	36955367	C	T	0.3171	0.6142
22	rs2235682	36983428	A	G	0.2192	0.8128
22	rs2283986	36989406	A	G	0.4423	0.9814
22	rs4821513	37013819	C	T	0.2095	0.7818
22	rs2283992	37015271	A	T	0.4070	0.9430
22	rs5756268	37020141	T	C	0.2259	0.9471
22	rs12628674	37063016	C	T	0.1756	0.9056
22	rs738516	37087493	C	A	0.2362	0.7748
22	rs2092662	37088339	G	T	0.2306	0.7689
22	rs2267361	37100260	A	G	0.4460	0.9814
19	rs11672982	54429198	T	G	0.2762	0.9315
19	rs10411229	54434126	T	G	0.3208	0.9788
19	rs11084305	54460053	T	G	0.2719	0.6428
19	rs10420331	54469068	G	A	0.3838	0.7334
19	rs11084307	54481150	T	C	0.4278	0.8875
19	rs192808	54510717	A	G	0.1095	0.5207
19	rs459247	54515874	C	T	0.4165	0.6525
17	rs2048137	64867456	G	T	0.1535	1.0000
17	rs740805	64871070	A	G	0.4258	0.9249
17	rs11659136	64878728	T	C	0.2558	0.8313
17	rs2286678	64881075	G	A	0.4438	0.9255
17	rs11079671	64886173	G	C	0.2228	0.9200
17	rs4791016	64887517	A	C	0.1445	0.8142
17	rs17645023	64917033	T	A	0.2470	0.8313
17	rs4790998	64988706	T	C	0.3554	0.8598
17	rs8071936	64999003	G	T	0.2941	0.9340
17	rs2885928	65035235	C	T	0.1102	0.9055
17	rs2159210	65035960	A	G	0.2694	0.8381
17	rs16960487	65041073	A	G	0.1145	0.8180
17	rs3785579	65042501	C	G	0.1751	0.9056
17	rs2363843	65045786	T	C	0.1203	0.4746

Supplemental Table S4. Full results of the single marker based association study. The three coding models were shown as "ADD" for additive, "DOM" for dominant and "REC" for recessive. Significant findings are highlighted in bold.

CHR	SNP	BP	A1	OR_allelic	STAT_ allelic	*P*_allelic	OR_ADD	STAT_ ADD	*P*_ADD	OR_DOM	STAT_ DOM	*P*_DOM	OR_REC	STAT_REC	*P*_REC
16	rs11646957	24255988	A	1.059	1.963	0.161	1.061	1.438	0.150	1.069	1.381	0.167	1.095	0.757	0.449
16	rs7204986	24301447	C	0.943	1.665	0.197	0.945	-1.254	0.210	0.951	-0.986	0.324	0.820	-1.273	0.203
16	rs4787985	24318774	T	1.052	1.773	0.183	1.052	1.329	0.184	1.056	1.162	0.245	1.102	0.982	0.326
16	rs11074616	24329370	A	0.961	0.888	0.346	0.961	-0.956	0.339	0.964	-0.763	0.445	0.890	-0.903	0.367
17	rs2048137	66871338	G	1.046	1.007	0.316	1.042	0.911	0.362	1.040	0.767	0.443	1.122	0.781	0.435
17	rs740805	66874952	A	0.980	0.362	0.547	0.976	-0.731	0.465	0.973	-0.561	0.575	0.962	-0.641	0.521
17	rs11659136	66882611	T	1.038	0.999	0.318	1.037	0.973	0.331	1.039	0.836	0.403	1.071	0.747	0.455
17	rs2286678	66884958	G	1.028	0.736	0.391	1.027	0.822	0.411	1.046	0.889	0.374	1.024	0.419	0.676
17	rs11079671	66890056	G	1.040	1.041	0.308	1.040	1.016	0.310	1.038	0.794	0.427	1.104	0.963	0.335
17	rs4791016	66891400	A	1.048	1.048	0.306	1.049	1.042	0.298	1.042	0.790	0.429	1.199	1.172	0.241
17	rs17645023	66920916	T	0.858	15.730	**7.30E-05**	0.856	-4.036	**5.43E-05**	0.846	-3.578	**3.46E-04**	0.743	-2.874	0.004
17	rs4790998	66992590	T	1.040	1.368	0.242	1.043	1.228	0.220	1.050	1.047	0.295	1.067	0.950	0.342
17	rs8071936	67002887	G	0.947	2.263	0.133	0.951	-1.392	0.164	0.946	-1.217	0.224	0.918	-1.024	0.306
17	rs2885928	67039119	C	1.050	0.894	0.344	1.050	0.945	0.345	1.043	0.744	0.457	1.230	1.053	0.293
17	rs2159210	67039844	A	0.971	0.649	0.421	0.969	-0.868	0.386	0.967	-0.729	0.466	0.939	-0.696	0.487
17	rs16960487	67044957	A	1.060	1.307	0.253	1.058	1.110	0.267	1.052	0.914	0.361	1.227	1.080	0.280
17	rs3785579	67046385	C	0.945	1.723	0.189	0.942	-1.368	0.171	0.943	-1.181	0.238	0.861	-1.069	0.285
17	rs2363843	67049670	T	1.049	0.930	0.335	1.048	0.948	0.343	1.039	0.700	0.484	1.231	1.176	0.240
19	rs11672982	53925944	T	0.945	2.345	0.126	0.944	-1.560	0.119	0.941	-1.323	0.186	0.897	-1.226	0.220
19	rs10411229	53930872	T	1.051	2.091	0.148	1.050	1.411	0.158	1.059	1.248	0.212	1.079	1.017	0.309
19	rs11084305	53956799	T	0.960	1.244	0.265	0.958	-1.157	0.247	0.958	-0.928	0.353	0.913	-1.009	0.313
19	rs10420331	53965814	G	0.963	1.264	0.261	0.963	-1.123	0.262	0.951	-1.068	0.285	0.954	-0.714	0.475
19	rs11084307	53977896	T	1.029	0.757	0.384	1.029	0.876	0.381	1.029	0.578	0.564	1.054	0.885	0.376
19	rs192808	54007463	A	0.940	1.383	0.240	0.943	-1.109	0.267	0.943	-1.016	0.310	0.857	-0.718	0.473
19	rs459247	54012620	C	0.976	0.558	0.455	0.979	-0.658	0.511	0.969	-0.644	0.520	0.976	-0.407	0.684

22	rs140034	36559320	C	0.964	1.082	0.298	0.966	-0.982	0.326	0.956	-0.987	0.324	0.961	-0.515	0.607
22	rs2235682	36587381	A	1.028	0.509	0.476	1.029	0.725	0.469	1.026	0.535	0.593	1.083	0.762	0.446
22	rs2283986	36593359	A	0.972	0.743	0.389	0.973	-0.825	0.409	0.964	-0.738	0.461	0.966	-0.599	0.549
22	rs4821513	36617772	C	0.963	0.862	0.353	0.964	-0.922	0.357	0.966	-0.729	0.466	0.905	-0.865	0.387
22	rs2283992	36619224	A	0.967	1.019	0.313	0.968	-0.965	0.335	0.952	-1.018	0.309	0.970	-0.496	0.620
22	rs5756268	36624094	T	1.045	1.299	0.255	1.046	1.171	0.242	1.052	1.088	0.277	1.078	0.724	0.469
22	rs12628674	36666969	C	0.960	0.918	0.338	0.959	-0.963	0.336	0.960	-0.832	0.405	0.902	-0.750	0.454
22	rs738516	36691448	C	0.961	1.043	0.307	0.961	-1.028	0.304	0.964	-0.788	0.431	0.902	-0.998	0.318
22	rs2092662	36692294	G	1.035	0.787	0.375	1.035	0.893	0.372	1.031	0.643	0.520	1.100	0.960	0.337
22	rs2267361	36704215	A	1.032	0.911	0.340	1.032	0.965	0.335	1.052	1.000	0.317	1.032	0.542	0.588

Supplemental Table S5. Full results of the two-way interaction analyses. "inter.*P*" indicates the theoretical *P* values and "perm.*P*" indicates the permutation *P* values. Significant findings are highlighted in bold.

SNP1	CHR1	GENE1	SNP2	CHR2	GENE2	inter.OR	inter.Z	inter.*P*	perm.*P*
rs2048137	17	CACNG5	rs192808	19	CACNG6	0.622	-4.675	**2.93E-06**	**<0.001**
rs740805	17	CACNG5	rs192808	19	CACNG6	0.766	-3.585	3.37E-04	0.104
rs7204986	16	CACNG3	rs459247	19	CACNG6	1.173	2.486	0.013	0.993
rs8071936	17	CACNG4	rs192808	19	CACNG6	1.222	2.473	0.013	0.993
rs10420331	19	CACNG8	rs2092662	22	CACNG2	1.145	2.461	0.014	0.994
rs4791016	17	CACNG5	rs2092662	22	CACNG2	1.204	2.448	0.014	0.994
rs11074616	16	CACNG3	rs738516	22	CACNG2	0.842	-2.414	0.016	0.997
rs11079671	17	CACNG5	rs2092662	22	CACNG2	1.165	2.400	0.016	0.998
rs11074616	16	CACNG3	rs2092662	22	CACNG2	0.845	-2.378	0.017	0.998
rs2286678	17	CACNG5	rs11672982	19	CACNG7	1.129	2.348	0.019	1.000
rs2048137	17	CACNG5	rs459247	19	CACNG6	0.865	-2.302	0.021	1.000
rs4787985	16	CACNG3	rs11084305	19	CACNG8	0.873	-2.204	0.028	1.000
rs11074616	16	CACNG3	rs16960487	17	CACNG1	0.813	-2.197	0.028	1.000
rs2159210	17	CACNG1	rs2267361	22	CACNG2	0.891	-2.192	0.028	1.000
rs17645023	17	CACNG5	rs5756268	22	CACNG2	0.866	-2.192	0.028	1.000
rs16960487	17	CACNG1	rs459247	19	CACNG6	1.168	2.181	0.029	1.000
rs10420331	19	CACNG8	rs738516	22	CACNG2	1.130	2.176	0.030	1.000
rs17645023	17	CACNG5	rs12628674	22	CACNG2	0.858	-2.091	0.036	1.000
rs11646957	16	CACNG3	rs140034	22	CACNG2	0.882	-2.046	0.041	1.000
rs11074616	16	CACNG3	rs2885928	17	CACNG1	0.824	-2.039	0.041	1.000
rs11074616	16	CACNG3	rs11659136	17	CACNG5	0.871	-2.023	0.043	1.000
rs8071936	17	CACNG4	rs12628674	22	CACNG2	0.871	-2.021	0.043	1.000
rs11079671	17	CACNG5	rs738516	22	CACNG2	1.138	2.012	0.044	1.000
rs4791016	17	CACNG5	rs738516	22	CACNG2	1.165	1.988	0.047	1.000
rs3785579	17	CACNG1	rs738516	22	CACNG2	1.154	1.981	0.048	1.000
rs8071936	17	CACNG4	rs2092662	22	CACNG2	0.890	-1.921	0.055	1.000
rs2159210	17	CACNG1	rs459247	19	CACNG6	1.107	1.902	0.057	1.000
rs8071936	17	CACNG4	rs2267361	22	CACNG2	0.907	-1.892	0.059	1.000
rs11646957	16	CACNG3	rs459247	19	CACNG6	1.119	1.884	0.060	1.000
rs11084307	19	CACNG8	rs2092662	22	CACNG2	1.108	1.879	0.060	1.000
rs11646957	16	CACNG3	rs5756268	22	CACNG2	1.134	1.874	0.061	1.000
rs3785579	17	CACNG1	rs2092662	22	CACNG2	1.145	1.873	0.061	1.000
rs17645023	17	CACNG5	rs11084307	19	CACNG8	0.901	-1.870	0.061	1.000
rs2363843	17	CACNG1	rs192808	19	CACNG6	1.224	1.862	0.063	1.000
rs8071936	17	CACNG4	rs738516	22	CACNG2	0.893	-1.851	0.064	1.000
rs16960487	17	CACNG1	rs140034	22	CACNG2	0.865	-1.847	0.065	1.000
rs4791016	17	CACNG5	rs459247	19	CACNG6	1.128	1.843	0.065	1.000
rs192808	19	CACNG6	rs2235682	22	CACNG2	1.168	1.841	0.066	1.000
rs11084307	19	CACNG8	rs738516	22	CACNG2	1.107	1.832	0.067	1.000

rs4787985	16	CACNG3	rs4790998	17	CACNG4	1.109	1.830	0.067	1.000
rs11079671	17	CACNG5	rs10411229	19	CACNG7	1.112	1.825	0.068	1.000
rs4791016	17	CACNG5	rs2267361	22	CACNG2	0.886	-1.823	0.068	1.000
rs11646957	16	CACNG3	rs2885928	17	CACNG1	1.179	1.801	0.072	1.000
rs2286678	17	CACNG5	rs2283986	22	CACNG2	1.086	1.775	0.076	1.000
rs11074616	16	CACNG3	rs3785579	17	CACNG1	0.867	-1.773	0.076	1.000
rs3785579	17	CACNG1	rs10411229	19	CACNG7	1.120	1.749	0.080	1.000
rs3785579	17	CACNG1	rs140034	22	CACNG2	0.889	-1.748	0.081	1.000
rs7204986	16	CACNG3	rs2235682	22	CACNG2	1.137	1.720	0.085	1.000
rs3785579	17	CACNG1	rs192808	19	CACNG6	1.185	1.713	0.087	1.000
rs16960487	17	CACNG1	rs2283986	22	CACNG2	0.884	-1.703	0.089	1.000
rs2286678	17	CACNG5	rs10420331	19	CACNG8	1.084	1.680	0.093	1.000
rs17645023	17	CACNG5	rs2283992	22	CACNG2	0.911	-1.677	0.094	1.000
rs8071936	17	CACNG4	rs459247	19	CACNG6	0.917	-1.675	0.094	1.000
rs2159210	17	CACNG1	rs5756268	22	CACNG2	0.902	-1.674	0.094	1.000
rs4791016	17	CACNG5	rs10411229	19	CACNG7	1.122	1.663	0.096	1.000
rs2048137	17	CACNG5	rs2267361	22	CACNG2	0.900	-1.649	0.099	1.000
rs459247	19	CACNG6	rs12628674	22	CACNG2	0.903	-1.645	0.100	1.000
rs740805	17	CACNG5	rs2267361	22	CACNG2	0.925	-1.644	0.100	1.000
rs11074616	16	CACNG3	rs2048137	17	CACNG5	0.871	-1.642	0.100	1.000
rs2286678	17	CACNG5	rs10411229	19	CACNG7	1.084	1.641	0.101	1.000
rs7204986	16	CACNG3	rs11659136	17	CACNG5	0.886	-1.628	0.104	1.000
rs2048137	17	CACNG5	rs11672982	19	CACNG7	0.888	-1.624	0.104	1.000
rs11079671	17	CACNG5	rs140034	22	CACNG2	1.099	1.617	0.106	1.000
rs7204986	16	CACNG3	rs2286678	17	CACNG5	0.900	-1.603	0.109	1.000
rs459247	19	CACNG6	rs2235682	22	CACNG2	0.913	-1.599	0.110	1.000
rs3785579	17	CACNG1	rs10420331	19	CACNG8	1.107	1.593	0.111	1.000
rs4790998	17	CACNG4	rs459247	19	CACNG6	0.926	-1.591	0.112	1.000
rs4787985	16	CACNG3	rs2286678	17	CACNG5	0.917	-1.584	0.113	1.000
rs4790998	17	CACNG4	rs192808	19	CACNG6	1.129	1.578	0.115	1.000
rs4787985	16	CACNG3	rs11659136	17	CACNG5	0.908	-1.573	0.116	1.000
rs11074616	16	CACNG3	rs192808	19	CACNG6	0.857	-1.559	0.119	1.000
rs2159210	17	CACNG1	rs12628674	22	CACNG2	0.900	-1.542	0.123	1.000
rs8071936	17	CACNG4	rs11084307	19	CACNG8	0.925	-1.517	0.129	1.000
rs11079671	17	CACNG5	rs10420331	19	CACNG8	1.089	1.512	0.130	1.000
rs3785579	17	CACNG1	rs2283986	22	CACNG2	0.912	-1.489	0.136	1.000
rs2048137	17	CACNG5	rs2092662	22	CACNG2	1.114	1.453	0.146	1.000
rs740805	17	CACNG5	rs2235682	22	CACNG2	0.921	-1.451	0.147	1.000
rs11079671	17	CACNG5	rs2283986	22	CACNG2	1.082	1.444	0.149	1.000
rs11079671	17	CACNG5	rs11084307	19	CACNG8	1.082	1.443	0.149	1.000
rs11079671	17	CACNG5	rs11672982	19	CACNG7	1.095	1.439	0.150	1.000
rs7204986	16	CACNG3	rs16960487	17	CACNG1	0.868	-1.433	0.152	1.000
rs10420331	19	CACNG8	rs12628674	22	CACNG2	1.092	1.423	0.155	1.000
rs2159210	17	CACNG1	rs2283992	22	CACNG2	0.929	-1.397	0.162	1.000
rs11074616	16	CACNG3	rs4791016	17	CACNG5	0.887	-1.391	0.164	1.000
rs4787985	16	CACNG3	rs2267361	22	CACNG2	0.928	-1.390	0.165	1.000

rs11646957	16	CACNG3	rs4821513	22	CACNG2	0.904	-1.384	0.166	1.000
rs3785579	17	CACNG1	rs2283992	22	CACNG2	1.089	1.376	0.169	1.000
rs4791016	17	CACNG5	rs11672982	19	CACNG7	1.108	1.370	0.171	1.000
rs11084305	19	CACNG8	rs140034	22	CACNG2	1.080	1.368	0.171	1.000
rs4790998	17	CACNG4	rs2235682	22	CACNG2	1.082	1.367	0.172	1.000
rs11079671	17	CACNG5	rs2235682	22	CACNG2	1.094	1.366	0.172	1.000
rs7204986	16	CACNG3	rs2283986	22	CACNG2	1.091	1.342	0.180	1.000
rs17645023	17	CACNG5	rs10420331	19	CACNG8	0.927	-1.333	0.183	1.000
rs459247	19	CACNG6	rs2283992	22	CACNG2	0.939	-1.331	0.183	1.000
rs11079671	17	CACNG5	rs459247	19	CACNG6	1.075	1.320	0.187	1.000
rs11074616	16	CACNG3	rs2286678	17	CACNG5	0.923	-1.319	0.187	1.000
rs2048137	17	CACNG5	rs738516	22	CACNG2	1.102	1.317	0.188	1.000
rs11672982	19	CACNG7	rs2092662	22	CACNG2	1.082	1.291	0.197	1.000
rs4787985	16	CACNG3	rs2885928	17	CACNG1	0.896	-1.290	0.197	1.000
rs4787985	16	CACNG3	rs5756268	22	CACNG2	0.921	-1.278	0.201	1.000
rs2885928	17	CACNG1	rs192808	19	CACNG6	0.859	-1.277	0.202	1.000
rs11672982	19	CACNG7	rs5756268	22	CACNG2	1.082	1.273	0.203	1.000
rs8071936	17	CACNG4	rs140034	22	CACNG2	0.932	-1.268	0.205	1.000
rs16960487	17	CACNG1	rs11672982	19	CACNG7	0.903	-1.249	0.212	1.000
rs17645023	17	CACNG5	rs459247	19	CACNG6	1.071	1.246	0.213	1.000
rs2286678	17	CACNG5	rs4821513	22	CACNG2	1.074	1.242	0.214	1.000
rs10411229	19	CACNG7	rs2283992	22	CACNG2	1.063	1.240	0.215	1.000
rs2286678	17	CACNG5	rs11084307	19	CACNG8	1.060	1.235	0.217	1.000
rs11079671	17	CACNG5	rs11084305	19	CACNG8	1.078	1.210	0.226	1.000
rs3785579	17	CACNG1	rs11084307	19	CACNG8	1.079	1.210	0.226	1.000
rs11074616	16	CACNG3	rs2159210	17	CACNG1	0.921	-1.207	0.227	1.000
rs4787985	16	CACNG3	rs10411229	19	CACNG7	1.073	1.206	0.228	1.000
rs11074616	16	CACNG3	rs12628674	22	CACNG2	0.910	-1.197	0.231	1.000
rs2363843	17	CACNG1	rs140034	22	CACNG2	0.914	-1.178	0.239	1.000
rs11646957	16	CACNG3	rs11084307	19	CACNG8	0.933	-1.176	0.240	1.000
rs7204986	16	CACNG3	rs4790998	17	CACNG4	1.080	1.168	0.243	1.000
rs2159210	17	CACNG1	rs2283986	22	CACNG2	0.940	-1.168	0.243	1.000
rs11084305	19	CACNG8	rs2283992	22	CACNG2	0.940	-1.163	0.245	1.000
rs4787985	16	CACNG3	rs12628674	22	CACNG2	0.921	-1.150	0.250	1.000
rs4791016	17	CACNG5	rs140034	22	CACNG2	1.083	1.145	0.252	1.000
rs16960487	17	CACNG1	rs10420331	19	CACNG8	1.088	1.144	0.252	1.000
rs4791016	17	CACNG5	rs11084305	19	CACNG8	1.088	1.143	0.253	1.000
rs2363843	17	CACNG1	rs10411229	19	CACNG7	1.089	1.138	0.255	1.000
rs11672982	19	CACNG7	rs12628674	22	CACNG2	1.081	1.135	0.256	1.000
rs11084305	19	CACNG8	rs2092662	22	CACNG2	1.073	1.132	0.258	1.000
rs10411229	19	CACNG7	rs5756268	22	CACNG2	1.068	1.123	0.261	1.000
rs7204986	16	CACNG3	rs11672982	19	CACNG7	1.084	1.111	0.267	1.000
rs4787985	16	CACNG3	rs2283986	22	CACNG2	0.941	-1.108	0.268	1.000
rs2159210	17	CACNG1	rs10420331	19	CACNG8	1.061	1.104	0.270	1.000
rs11074616	16	CACNG3	rs17645023	17	CACNG5	0.925	-1.100	0.271	1.000
rs4787985	16	CACNG3	rs2092662	22	CACNG2	0.932	-1.098	0.272	1.000

rs16960487	17	CACNG1	rs11084307	19	CACNG8	1.083	1.093	0.274	1.000
rs11659136	17	CACNG5	rs2235682	22	CACNG2	1.070	1.091	0.275	1.000
rs11672982	19	CACNG7	rs4821513	22	CACNG2	0.932	-1.089	0.276	1.000
rs740805	17	CACNG5	rs459247	19	CACNG6	0.950	-1.088	0.277	1.000
rs4791016	17	CACNG5	rs12628674	22	CACNG2	1.097	1.084	0.278	1.000
rs11084305	19	CACNG8	rs4821513	22	CACNG2	0.933	-1.066	0.286	1.000
rs11074616	16	CACNG3	rs5756268	22	CACNG2	0.928	-1.064	0.287	1.000
rs11659136	17	CACNG5	rs10420331	19	CACNG8	1.058	1.052	0.293	1.000
rs11646957	16	CACNG3	rs17645023	17	CACNG5	1.073	1.040	0.299	1.000
rs459247	19	CACNG6	rs5756268	22	CACNG2	0.945	-1.027	0.305	1.000
rs192808	19	CACNG6	rs12628674	22	CACNG2	0.904	-1.012	0.312	1.000
rs11646957	16	CACNG3	rs11084305	19	CACNG8	0.937	-1.006	0.315	1.000
rs10411229	19	CACNG7	rs2235682	22	CACNG2	1.061	0.995	0.320	1.000
rs2159210	17	CACNG1	rs10411229	19	CACNG7	0.948	-0.983	0.326	1.000
rs3785579	17	CACNG1	rs2235682	22	CACNG2	0.929	-0.980	0.327	1.000
rs2885928	17	CACNG1	rs11084307	19	CACNG8	1.074	0.975	0.329	1.000
rs2159210	17	CACNG1	rs140034	22	CACNG2	1.056	0.974	0.330	1.000
rs2363843	17	CACNG1	rs2235682	22	CACNG2	1.083	0.964	0.335	1.000
rs2363843	17	CACNG1	rs11084307	19	CACNG8	1.069	0.959	0.337	1.000
rs7204986	16	CACNG3	rs4821513	22	CACNG2	1.079	0.959	0.338	1.000
rs11079671	17	CACNG5	rs12628674	22	CACNG2	1.071	0.953	0.340	1.000
rs459247	19	CACNG6	rs2283986	22	CACNG2	0.956	-0.951	0.341	1.000
rs11646957	16	CACNG3	rs2092662	22	CACNG2	0.936	-0.947	0.344	1.000
rs4790998	17	CACNG4	rs738516	22	CACNG2	0.949	-0.945	0.344	1.000
rs17645023	17	CACNG5	rs11672982	19	CACNG7	0.944	-0.945	0.345	1.000
rs740805	17	CACNG5	rs11084307	19	CACNG8	0.956	-0.940	0.347	1.000
rs8071936	17	CACNG4	rs11084305	19	CACNG8	0.948	-0.937	0.349	1.000
rs2363843	17	CACNG1	rs2283992	22	CACNG2	1.068	0.931	0.352	1.000
rs2048137	17	CACNG5	rs2235682	22	CACNG2	0.931	-0.927	0.354	1.000
rs17645023	17	CACNG5	rs2235682	22	CACNG2	0.940	-0.922	0.357	1.000
rs11659136	17	CACNG5	rs11672982	19	CACNG7	1.055	0.920	0.357	1.000
rs192808	19	CACNG6	rs2283992	22	CACNG2	0.933	-0.911	0.362	1.000
rs11079671	17	CACNG5	rs2267361	22	CACNG2	0.951	-0.908	0.364	1.000
rs10411229	19	CACNG7	rs4821513	22	CACNG2	1.058	0.905	0.365	1.000
rs192808	19	CACNG6	rs738516	22	CACNG2	0.924	-0.885	0.376	1.000
rs10411229	19	CACNG7	rs738516	22	CACNG2	0.950	-0.883	0.377	1.000
rs10420331	19	CACNG8	rs5756268	22	CACNG2	1.050	0.882	0.378	1.000
rs740805	17	CACNG5	rs10420331	19	CACNG8	0.958	-0.881	0.378	1.000
rs11079671	17	CACNG5	rs192808	19	CACNG6	0.925	-0.876	0.381	1.000
rs2286678	17	CACNG5	rs11084305	19	CACNG8	1.047	0.871	0.384	1.000
rs10420331	19	CACNG8	rs4821513	22	CACNG2	0.950	-0.867	0.386	1.000
rs2286678	17	CACNG5	rs2283992	22	CACNG2	1.041	0.859	0.391	1.000
rs16960487	17	CACNG1	rs4821513	22	CACNG2	0.927	-0.853	0.393	1.000
rs11074616	16	CACNG3	rs2267361	22	CACNG2	0.950	-0.850	0.395	1.000
rs17645023	17	CACNG5	rs2267361	22	CACNG2	0.954	-0.848	0.396	1.000
rs2363843	17	CACNG1	rs2283986	22	CACNG2	0.942	-0.848	0.396	1.000

rs8071936	17	CACNG4	rs11672982	19	CACNG7	0.952	-0.846	0.398	1.000
rs2159210	17	CACNG1	rs192808	19	CACNG6	1.074	0.844	0.399	1.000
rs7204986	16	CACNG3	rs2885928	17	CACNG1	0.918	-0.842	0.400	1.000
rs4787985	16	CACNG3	rs2159210	17	CACNG1	0.950	-0.842	0.400	1.000
rs4790998	17	CACNG4	rs10411229	19	CACNG7	0.958	-0.835	0.404	1.000
rs4787985	16	CACNG3	rs140034	22	CACNG2	0.952	-0.834	0.404	1.000
rs459247	19	CACNG6	rs738516	22	CACNG2	0.955	-0.833	0.405	1.000
rs11646957	16	CACNG3	rs2286678	17	CACNG5	0.953	-0.827	0.408	1.000
rs11646957	16	CACNG3	rs192808	19	CACNG6	1.081	0.824	0.410	1.000
rs4791016	17	CACNG5	rs2283986	22	CACNG2	1.055	0.813	0.416	1.000
rs4791016	17	CACNG5	rs2235682	22	CACNG2	1.064	0.809	0.418	1.000
rs11672982	19	CACNG7	rs2283986	22	CACNG2	0.959	-0.808	0.419	1.000
rs2048137	17	CACNG5	rs2283986	22	CACNG2	1.052	0.803	0.422	1.000
rs11074616	16	CACNG3	rs4790998	17	CACNG4	1.050	0.798	0.425	1.000
rs8071936	17	CACNG4	rs10411229	19	CACNG7	0.957	-0.792	0.428	1.000
rs459247	19	CACNG6	rs2092662	22	CACNG2	0.958	-0.786	0.432	1.000
rs11074616	16	CACNG3	rs2363843	17	CACNG1	0.932	-0.785	0.433	1.000
rs4790998	17	CACNG4	rs10420331	19	CACNG8	1.040	0.782	0.434	1.000
rs11646957	16	CACNG3	rs2283992	22	CACNG2	1.047	0.778	0.436	1.000
rs8071936	17	CACNG4	rs2283986	22	CACNG2	0.961	-0.778	0.436	1.000
rs192808	19	CACNG6	rs2092662	22	CACNG2	0.934	-0.777	0.437	1.000
rs11074616	16	CACNG3	rs11079671	17	CACNG5	0.946	-0.770	0.442	1.000
rs192808	19	CACNG6	rs140034	22	CACNG2	1.063	0.769	0.442	1.000
rs4791016	17	CACNG5	rs5756268	22	CACNG2	0.943	-0.766	0.444	1.000
rs11074616	16	CACNG3	rs11672982	19	CACNG7	0.950	-0.765	0.444	1.000
rs7204986	16	CACNG3	rs740805	17	CACNG5	1.050	0.765	0.444	1.000
rs2048137	17	CACNG5	rs10411229	19	CACNG7	0.950	-0.762	0.446	1.000
rs2885928	17	CACNG1	rs11084305	19	CACNG8	1.063	0.761	0.447	1.000
rs2885928	17	CACNG1	rs5756268	22	CACNG2	0.935	-0.753	0.451	1.000
rs11079671	17	CACNG5	rs4821513	22	CACNG2	0.950	-0.743	0.457	1.000
rs17645023	17	CACNG5	rs738516	22	CACNG2	0.953	-0.731	0.465	1.000
rs4790998	17	CACNG4	rs11084305	19	CACNG8	0.961	-0.729	0.466	1.000
rs2885928	17	CACNG1	rs12628674	22	CACNG2	0.931	-0.727	0.467	1.000
rs11084307	19	CACNG8	rs140034	22	CACNG2	1.037	0.726	0.468	1.000
rs4791016	17	CACNG5	rs11084307	19	CACNG8	1.049	0.725	0.468	1.000
rs2048137	17	CACNG5	rs4821513	22	CACNG2	1.058	0.721	0.471	1.000
rs740805	17	CACNG5	rs5756268	22	CACNG2	0.960	-0.721	0.471	1.000
rs4787985	16	CACNG3	rs738516	22	CACNG2	0.954	-0.720	0.472	1.000
rs2885928	17	CACNG1	rs4821513	22	CACNG2	0.938	-0.713	0.476	1.000
rs11646957	16	CACNG3	rs12628674	22	CACNG2	1.058	0.713	0.476	1.000
rs4790998	17	CACNG4	rs5756268	22	CACNG2	1.041	0.712	0.476	1.000
rs11672982	19	CACNG7	rs738516	22	CACNG2	1.044	0.699	0.485	1.000
rs16960487	17	CACNG1	rs2092662	22	CACNG2	1.061	0.698	0.485	1.000
rs2048137	17	CACNG5	rs10420331	19	CACNG8	0.955	-0.698	0.485	1.000
rs10420331	19	CACNG8	rs2283992	22	CACNG2	1.034	0.687	0.492	1.000
rs11084305	19	CACNG8	rs12628674	22	CACNG2	1.048	0.684	0.494	1.000

rs11084305	19	CACNG8	rs738516	22	CACNG2	1.043	0.682	0.495	1.000
rs11646957	16	CACNG3	rs2283986	22	CACNG2	1.040	0.682	0.495	1.000
rs11084307	19	CACNG8	rs12628674	22	CACNG2	1.043	0.681	0.496	1.000
rs11074616	16	CACNG3	rs2283986	22	CACNG2	0.960	-0.678	0.498	1.000
rs17645023	17	CACNG5	rs11084305	19	CACNG8	1.042	0.677	0.498	1.000
rs16960487	17	CACNG1	rs11084305	19	CACNG8	1.055	0.675	0.499	1.000
rs11084307	19	CACNG8	rs4821513	22	CACNG2	0.962	-0.675	0.500	1.000
rs10411229	19	CACNG7	rs12628674	22	CACNG2	1.045	0.673	0.501	1.000
rs2286678	17	CACNG5	rs5756268	22	CACNG2	1.037	0.669	0.503	1.000
rs4790998	17	CACNG4	rs11672982	19	CACNG7	0.965	-0.662	0.508	1.000
rs2885928	17	CACNG1	rs459247	19	CACNG6	0.954	-0.660	0.510	1.000
rs11646957	16	CACNG3	rs2235682	22	CACNG2	0.955	-0.659	0.510	1.000
rs11074616	16	CACNG3	rs2283992	22	CACNG2	1.041	0.657	0.511	1.000
rs2286678	17	CACNG5	rs2235682	22	CACNG2	1.037	0.651	0.515	1.000
rs8071936	17	CACNG4	rs2283992	22	CACNG2	0.967	-0.648	0.517	1.000
rs11084305	19	CACNG8	rs2267361	22	CACNG2	0.967	-0.635	0.525	1.000
rs8071936	17	CACNG4	rs10420331	19	CACNG8	0.967	-0.633	0.527	1.000
rs11646957	16	CACNG3	rs10420331	19	CACNG8	0.962	-0.632	0.527	1.000
rs11646957	16	CACNG3	rs738516	22	CACNG2	0.957	-0.632	0.528	1.000
rs4790998	17	CACNG4	rs140034	22	CACNG2	0.968	-0.631	0.528	1.000
rs11074616	16	CACNG3	rs740805	17	CACNG5	0.964	-0.627	0.530	1.000
rs11074616	16	CACNG3	rs2235682	22	CACNG2	0.955	-0.621	0.534	1.000
rs17645023	17	CACNG5	rs4821513	22	CACNG2	1.042	0.617	0.537	1.000
rs4787985	16	CACNG3	rs2235682	22	CACNG2	0.960	-0.615	0.539	1.000
rs7204986	16	CACNG3	rs2159210	17	CACNG1	0.957	-0.612	0.540	1.000
rs11084305	19	CACNG8	rs2235682	22	CACNG2	0.962	-0.603	0.547	1.000
rs11646957	16	CACNG3	rs11079671	17	CACNG5	1.043	0.603	0.547	1.000
rs17645023	17	CACNG5	rs10411229	19	CACNG7	0.966	-0.597	0.550	1.000
rs11074616	16	CACNG3	rs10411229	19	CACNG7	0.963	-0.596	0.551	1.000
rs17645023	17	CACNG5	rs192808	19	CACNG6	0.948	-0.592	0.554	1.000
rs4791016	17	CACNG5	rs2283992	22	CACNG2	0.962	-0.591	0.555	1.000
rs11659136	17	CACNG5	rs459247	19	CACNG6	0.969	-0.584	0.559	1.000
rs11659136	17	CACNG5	rs192808	19	CACNG6	0.951	-0.579	0.563	1.000
rs10411229	19	CACNG7	rs2283986	22	CACNG2	0.971	-0.578	0.563	1.000
rs192808	19	CACNG6	rs2283986	22	CACNG2	1.044	0.577	0.564	1.000
rs11074616	16	CACNG3	rs8071936	17	CACNG4	1.038	0.573	0.567	1.000
rs3785579	17	CACNG1	rs11672982	19	CACNG7	1.040	0.569	0.569	1.000
rs11084307	19	CACNG8	rs2283992	22	CACNG2	1.027	0.569	0.569	1.000
rs11646957	16	CACNG3	rs11659136	17	CACNG5	0.963	-0.564	0.573	1.000
rs4787985	16	CACNG3	rs192808	19	CACNG6	1.050	0.563	0.574	1.000
rs11646957	16	CACNG3	rs740805	17	CACNG5	1.033	0.561	0.575	1.000
rs2159210	17	CACNG1	rs11084305	19	CACNG8	1.033	0.551	0.581	1.000
rs4790998	17	CACNG4	rs2092662	22	CACNG2	0.970	-0.548	0.584	1.000
rs7204986	16	CACNG3	rs4791016	17	CACNG5	1.050	0.545	0.586	1.000
rs11659136	17	CACNG5	rs738516	22	CACNG2	0.966	-0.544	0.587	1.000
rs2286678	17	CACNG5	rs140034	22	CACNG2	1.027	0.542	0.588	1.000

rs2159210	17	CACNG1	rs11672982	19	CACNG7	0.968	-0.542	0.588	1.000
rs4787985	16	CACNG3	rs16960487	17	CACNG1	0.956	-0.538	0.591	1.000
rs4787985	16	CACNG3	rs17645023	17	CACNG5	0.966	-0.531	0.596	1.000
rs7204986	16	CACNG3	rs2363843	17	CACNG1	0.951	-0.527	0.598	1.000
rs11646957	16	CACNG3	rs2267361	22	CACNG2	1.031	0.524	0.600	1.000
rs11672982	19	CACNG7	rs140034	22	CACNG2	0.971	-0.520	0.603	1.000
rs4787985	16	CACNG3	rs459247	19	CACNG6	1.029	0.519	0.604	1.000
rs7204986	16	CACNG3	rs11084305	19	CACNG8	0.963	-0.515	0.607	1.000
rs4791016	17	CACNG5	rs4821513	22	CACNG2	0.960	-0.509	0.611	1.000
rs16960487	17	CACNG1	rs5756268	22	CACNG2	0.958	-0.506	0.613	1.000
rs11646957	16	CACNG3	rs4791016	17	CACNG5	1.042	0.504	0.614	1.000
rs740805	17	CACNG5	rs2283992	22	CACNG2	0.977	-0.496	0.620	1.000
rs2885928	17	CACNG1	rs140034	22	CACNG2	0.962	-0.493	0.622	1.000
rs11084305	19	CACNG8	rs2283986	22	CACNG2	1.026	0.490	0.624	1.000
rs17645023	17	CACNG5	rs2092662	22	CACNG2	0.969	-0.489	0.625	1.000
rs10420331	19	CACNG8	rs140034	22	CACNG2	1.024	0.471	0.637	1.000
rs2048137	17	CACNG5	rs2283992	22	CACNG2	0.970	-0.471	0.638	1.000
rs740805	17	CACNG5	rs2092662	22	CACNG2	1.026	0.468	0.640	1.000
rs7204986	16	CACNG3	rs8071936	17	CACNG4	0.968	-0.466	0.641	1.000
rs10420331	19	CACNG8	rs2283986	22	CACNG2	1.023	0.466	0.641	1.000
rs2363843	17	CACNG1	rs459247	19	CACNG6	1.033	0.463	0.643	1.000
rs2363843	17	CACNG1	rs10420331	19	CACNG8	1.034	0.462	0.644	1.000
rs11659136	17	CACNG5	rs2283986	22	CACNG2	1.025	0.462	0.644	1.000
rs11074616	16	CACNG3	rs4821513	22	CACNG2	0.966	-0.462	0.644	1.000
rs4787985	16	CACNG3	rs740805	17	CACNG5	1.025	0.460	0.645	1.000
rs11084307	19	CACNG8	rs5756268	22	CACNG2	1.025	0.455	0.649	1.000
rs2286678	17	CACNG5	rs12628674	22	CACNG2	1.028	0.450	0.652	1.000
rs2159210	17	CACNG1	rs11084307	19	CACNG8	1.024	0.447	0.655	1.000
rs11084305	19	CACNG8	rs5756268	22	CACNG2	0.973	-0.438	0.662	1.000
rs11672982	19	CACNG7	rs2283992	22	CACNG2	1.024	0.437	0.662	1.000
rs2048137	17	CACNG5	rs11084305	19	CACNG8	1.032	0.437	0.662	1.000
rs11646957	16	CACNG3	rs2363843	17	CACNG1	1.039	0.436	0.663	1.000
rs16960487	17	CACNG1	rs738516	22	CACNG2	1.038	0.436	0.663	1.000
rs2885928	17	CACNG1	rs2283992	22	CACNG2	0.968	-0.425	0.671	1.000
rs3785579	17	CACNG1	rs5756268	22	CACNG2	1.031	0.424	0.671	1.000
rs2363843	17	CACNG1	rs12628674	22	CACNG2	0.963	-0.418	0.676	1.000
rs4787985	16	CACNG3	rs4821513	22	CACNG2	0.972	-0.417	0.677	1.000
rs4790998	17	CACNG4	rs2267361	22	CACNG2	0.980	-0.417	0.677	1.000
rs11079671	17	CACNG5	rs2283992	22	CACNG2	1.023	0.413	0.680	1.000
rs7204986	16	CACNG3	rs192808	19	CACNG6	1.043	0.409	0.682	1.000
rs8071936	17	CACNG4	rs5756268	22	CACNG2	0.976	-0.400	0.689	1.000
rs4787985	16	CACNG3	rs4791016	17	CACNG5	0.970	-0.399	0.690	1.000
rs7204986	16	CACNG3	rs10420331	19	CACNG8	1.027	0.398	0.690	1.000
rs7204986	16	CACNG3	rs10411229	19	CACNG7	1.028	0.397	0.691	1.000
rs10411229	19	CACNG7	rs140034	22	CACNG2	1.020	0.389	0.697	1.000
rs16960487	17	CACNG1	rs2267361	22	CACNG2	0.973	-0.385	0.700	1.000

rs4787985	16	CACNG3	rs11084307	19	CACNG8	0.979	-0.385	0.700	1.000
rs192808	19	CACNG6	rs4821513	22	CACNG2	0.966	-0.377	0.706	1.000
rs11646957	16	CACNG3	rs4790998	17	CACNG4	1.023	0.375	0.708	1.000
rs2048137	17	CACNG5	rs5756268	22	CACNG2	0.972	-0.375	0.708	1.000
rs3785579	17	CACNG1	rs12628674	22	CACNG2	0.971	-0.373	0.710	1.000
rs3785579	17	CACNG1	rs2267361	22	CACNG2	0.978	-0.365	0.715	1.000
rs7204986	16	CACNG3	rs2092662	22	CACNG2	0.972	-0.364	0.716	1.000
rs7204986	16	CACNG3	rs11084307	19	CACNG8	1.023	0.355	0.722	1.000
rs11646957	16	CACNG3	rs16960487	17	CACNG1	0.969	-0.351	0.726	1.000
rs2159210	17	CACNG1	rs4821513	22	CACNG2	0.978	-0.349	0.727	1.000
rs4791016	17	CACNG5	rs10420331	19	CACNG8	1.023	0.340	0.734	1.000
rs2885928	17	CACNG1	rs2235682	22	CACNG2	0.972	-0.334	0.738	1.000
rs11659136	17	CACNG5	rs2092662	22	CACNG2	0.980	-0.331	0.741	1.000
rs11646957	16	CACNG3	rs10411229	19	CACNG7	1.021	0.330	0.742	1.000
rs2363843	17	CACNG1	rs2267361	22	CACNG2	1.024	0.329	0.742	1.000
rs11084307	19	CACNG8	rs2267361	22	CACNG2	1.016	0.329	0.742	1.000
rs192808	19	CACNG6	rs2267361	22	CACNG2	1.024	0.328	0.743	1.000
rs17645023	17	CACNG5	rs140034	22	CACNG2	0.981	-0.326	0.745	1.000
rs16960487	17	CACNG1	rs192808	19	CACNG6	1.037	0.321	0.748	1.000
rs3785579	17	CACNG1	rs11084305	19	CACNG8	1.023	0.321	0.748	1.000
rs3785579	17	CACNG1	rs4821513	22	CACNG2	1.024	0.320	0.749	1.000
rs11659136	17	CACNG5	rs2267361	22	CACNG2	1.017	0.316	0.752	1.000
rs7204986	16	CACNG3	rs2283992	22	CACNG2	1.021	0.316	0.752	1.000
rs10411229	19	CACNG7	rs2092662	22	CACNG2	0.982	-0.313	0.754	1.000
rs459247	19	CACNG6	rs4821513	22	CACNG2	0.982	-0.312	0.755	1.000
rs11659136	17	CACNG5	rs5756268	22	CACNG2	1.019	0.303	0.762	1.000
rs2159210	17	CACNG1	rs2235682	22	CACNG2	1.019	0.302	0.763	1.000
rs2363843	17	CACNG1	rs11084305	19	CACNG8	1.024	0.299	0.765	1.000
rs2159210	17	CACNG1	rs2092662	22	CACNG2	0.982	-0.296	0.767	1.000
rs11084307	19	CACNG8	rs2283986	22	CACNG2	0.986	-0.294	0.769	1.000
rs2885928	17	CACNG1	rs10411229	19	CACNG7	0.978	-0.293	0.769	1.000
rs459247	19	CACNG6	rs140034	22	CACNG2	0.986	-0.290	0.772	1.000
rs4787985	16	CACNG3	rs10420331	19	CACNG8	0.984	-0.289	0.773	1.000
rs4790998	17	CACNG4	rs12628674	22	CACNG2	0.982	-0.288	0.774	1.000
rs11074616	16	CACNG3	rs11084307	19	CACNG8	1.017	0.285	0.776	1.000
rs2885928	17	CACNG1	rs2283986	22	CACNG2	0.979	-0.284	0.776	1.000
rs7204986	16	CACNG3	rs12628674	22	CACNG2	1.024	0.278	0.781	1.000
rs2885928	17	CACNG1	rs2267361	22	CACNG2	0.980	-0.273	0.785	1.000
rs7204986	16	CACNG3	rs3785579	17	CACNG1	0.978	-0.271	0.786	1.000
rs4787985	16	CACNG3	rs8071936	17	CACNG4	1.016	0.260	0.795	1.000
rs8071936	17	CACNG4	rs2235682	22	CACNG2	0.984	-0.259	0.796	1.000
rs2363843	17	CACNG1	rs2092662	22	CACNG2	0.980	-0.245	0.807	1.000
rs2885928	17	CACNG1	rs11672982	19	CACNG7	1.020	0.244	0.807	1.000
rs3785579	17	CACNG1	rs459247	19	CACNG6	1.015	0.242	0.809	1.000
rs11659136	17	CACNG5	rs4821513	22	CACNG2	1.016	0.240	0.810	1.000
rs11646957	16	CACNG3	rs8071936	17	CACNG4	1.015	0.239	0.811	1.000

rs7204986	16	CACNG3	rs140034	22	CACNG2	1.016	0.239	0.811	1.000
rs2363843	17	CACNG1	rs5756268	22	CACNG2	1.020	0.237	0.813	1.000
rs2363843	17	CACNG1	rs4821513	22	CACNG2	1.021	0.236	0.813	1.000
rs7204986	16	CACNG3	rs2048137	17	CACNG5	1.020	0.227	0.821	1.000
rs4791016	17	CACNG5	rs192808	19	CACNG6	0.977	-0.223	0.824	1.000
rs740805	17	CACNG5	rs11672982	19	CACNG7	1.012	0.220	0.826	1.000
rs11074616	16	CACNG3	rs11084305	19	CACNG8	0.985	-0.218	0.827	1.000
rs11074616	16	CACNG3	rs10420331	19	CACNG8	0.987	-0.217	0.828	1.000
rs740805	17	CACNG5	rs2283986	22	CACNG2	1.010	0.216	0.829	1.000
rs7204986	16	CACNG3	rs11079671	17	CACNG5	0.984	-0.209	0.835	1.000
rs7204986	16	CACNG3	rs5756268	22	CACNG2	0.986	-0.192	0.848	1.000
rs16960487	17	CACNG1	rs2235682	22	CACNG2	1.016	0.192	0.848	1.000
rs2286678	17	CACNG5	rs192808	19	CACNG6	0.986	-0.185	0.853	1.000
rs459247	19	CACNG6	rs2267361	22	CACNG2	1.009	0.182	0.855	1.000
rs11646957	16	CACNG3	rs2048137	17	CACNG5	0.986	-0.182	0.856	1.000
rs4787985	16	CACNG3	rs11079671	17	CACNG5	0.988	-0.179	0.858	1.000
rs11646957	16	CACNG3	rs2159210	17	CACNG1	0.988	-0.175	0.861	1.000
rs11646957	16	CACNG3	rs11672982	19	CACNG7	1.012	0.175	0.861	1.000
rs7204986	16	CACNG3	rs17645023	17	CACNG5	0.987	-0.173	0.863	1.000
rs11079671	17	CACNG5	rs5756268	22	CACNG2	1.011	0.165	0.869	1.000
rs192808	19	CACNG6	rs5756268	22	CACNG2	1.015	0.163	0.870	1.000
rs11659136	17	CACNG5	rs140034	22	CACNG2	0.991	-0.157	0.876	1.000
rs2363843	17	CACNG1	rs738516	22	CACNG2	1.012	0.148	0.882	1.000
rs4787985	16	CACNG3	rs3785579	17	CACNG1	0.989	-0.148	0.882	1.000
rs740805	17	CACNG5	rs12628674	22	CACNG2	0.991	-0.147	0.883	1.000
rs11672982	19	CACNG7	rs2235682	22	CACNG2	0.991	-0.140	0.889	1.000
rs16960487	17	CACNG1	rs10411229	19	CACNG7	1.011	0.138	0.890	1.000
rs4790998	17	CACNG4	rs11084307	19	CACNG8	1.006	0.132	0.895	1.000
rs4790998	17	CACNG4	rs2283986	22	CACNG2	0.994	-0.127	0.899	1.000
rs11659136	17	CACNG5	rs11084307	19	CACNG8	1.007	0.126	0.900	1.000
rs2048137	17	CACNG5	rs140034	22	CACNG2	0.992	-0.113	0.910	1.000
rs11659136	17	CACNG5	rs11084305	19	CACNG8	1.007	0.112	0.911	1.000
rs4787985	16	CACNG3	rs2283992	22	CACNG2	1.006	0.111	0.912	1.000
rs2885928	17	CACNG1	rs10420331	19	CACNG8	1.008	0.108	0.914	1.000
rs11646957	16	CACNG3	rs3785579	17	CACNG1	1.008	0.107	0.915	1.000
rs740805	17	CACNG5	rs10411229	19	CACNG7	0.995	-0.105	0.916	1.000
rs740805	17	CACNG5	rs738516	22	CACNG2	1.006	0.104	0.917	1.000
rs2885928	17	CACNG1	rs2092662	22	CACNG2	1.009	0.101	0.919	1.000
rs2048137	17	CACNG5	rs11084307	19	CACNG8	0.994	-0.100	0.920	1.000
rs11659136	17	CACNG5	rs12628674	22	CACNG2	0.993	-0.098	0.922	1.000
rs4787985	16	CACNG3	rs2048137	17	CACNG5	0.993	-0.098	0.922	1.000
rs10420331	19	CACNG8	rs2267361	22	CACNG2	0.995	-0.094	0.925	1.000
rs7204986	16	CACNG3	rs2267361	22	CACNG2	1.006	0.090	0.928	1.000
rs10420331	19	CACNG8	rs2235682	22	CACNG2	1.005	0.087	0.930	1.000
rs2159210	17	CACNG1	rs738516	22	CACNG2	0.995	-0.085	0.932	1.000
rs4787985	16	CACNG3	rs2363843	17	CACNG1	0.993	-0.082	0.934	1.000

rs2286678	17	CACNG5	rs2267361	22	CACNG2	1.004	0.082	0.935	1.000
rs17645023	17	CACNG5	rs2283986	22	CACNG2	0.996	-0.078	0.938	1.000
rs740805	17	CACNG5	rs4821513	22	CACNG2	0.996	-0.077	0.939	1.000
rs2286678	17	CACNG5	rs2092662	22	CACNG2	1.004	0.073	0.942	1.000
rs4790998	17	CACNG4	rs4821513	22	CACNG2	1.004	0.071	0.944	1.000
rs7204986	16	CACNG3	rs738516	22	CACNG2	0.995	-0.068	0.946	1.000
rs2048137	17	CACNG5	rs12628674	22	CACNG2	1.006	0.067	0.947	1.000
rs11672982	19	CACNG7	rs2267361	22	CACNG2	0.997	-0.065	0.948	1.000
rs740805	17	CACNG5	rs11084305	19	CACNG8	1.003	0.061	0.951	1.000
rs8071936	17	CACNG4	rs4821513	22	CACNG2	1.004	0.057	0.955	1.000
rs4790998	17	CACNG4	rs2283992	22	CACNG2	1.003	0.056	0.955	1.000
rs11074616	16	CACNG3	rs140034	22	CACNG2	1.003	0.052	0.959	1.000
rs2286678	17	CACNG5	rs738516	22	CACNG2	1.003	0.050	0.960	1.000
rs11084307	19	CACNG8	rs2235682	22	CACNG2	0.998	-0.044	0.965	1.000
rs2363843	17	CACNG1	rs11672982	19	CACNG7	1.003	0.041	0.967	1.000
rs2286678	17	CACNG5	rs459247	19	CACNG6	1.002	0.040	0.968	1.000
rs740805	17	CACNG5	rs140034	22	CACNG2	0.998	-0.039	0.969	1.000
rs11659136	17	CACNG5	rs10411229	19	CACNG7	1.002	0.037	0.970	1.000
rs2885928	17	CACNG1	rs738516	22	CACNG2	0.997	-0.037	0.971	1.000
rs11074616	16	CACNG3	rs459247	19	CACNG6	1.002	0.032	0.975	1.000
rs10411229	19	CACNG7	rs2267361	22	CACNG2	1.002	0.031	0.976	1.000
rs16960487	17	CACNG1	rs2283992	22	CACNG2	0.998	-0.030	0.976	1.000
rs4787985	16	CACNG3	rs11672982	19	CACNG7	0.999	-0.017	0.987	1.000
rs11659136	17	CACNG5	rs2283992	22	CACNG2	0.999	-0.014	0.989	1.000
rs16960487	17	CACNG1	rs12628674	22	CACNG2	1.001	0.008	0.993	1.000

Supplemental Table S6. Full results of the association tests based on both genotyped and imputed SNPs. Significant findings are highlighted in bold.

SNP	POS	Allele A	Allele B	MAF	OR	*P*-value	Type
rs8071471	62282811	A	G	0.1545	0.9979	0.9133	Imputed
rs11868854	62288833	A	G	0.1605	0.9983	0.9784	Imputed
rs1120577	62296918	A	G	0.2176	1.0454	0.1425	Imputed
rs2048137	62297918	G	T	0.1535	0.9560	0.3623	Genotyped
rs740805	62301532	A	G	0.4258	1.0200	0.4648	Genotyped
rs758664	62301734	A	G	0.3335	0.9725	0.2958	Imputed
rs3760266	62301840	A	G	0.0749	0.9666	0.5438	Imputed
rs3760265	62302183	C	T	0.1560	1.0066	0.8993	Imputed
rs722195	62302531	C	T	0.0592	1.0321	0.5231	Imputed
rs11651303	62303385	A	G	0.0716	0.9939	0.9123	Imputed
rs1002327	62305077	C	T	0.3942	0.9903	0.7874	Imputed
rs2286676	62305497	C	T	0.3076	1.0151	0.7105	Imputed
rs11659136	62309190	C	T	0.2558	1.0378	0.3306	Genotyped
rs2286677	62311250	A	G	0.0611	1.0241	0.6186	Imputed
rs2286678	62311537	A	G	0.4438	1.0285	0.4108	Genotyped
rs9906660	62313188	C	T	0.1763	1.0629	0.0524	Imputed
rs9303512	62314129	A	G	0.1755	1.0492	0.1430	Imputed
rs9901824	62315913	A	C	0.2628	0.9790	0.5243	Imputed
rs11079671	62316635	C	G	0.2228	1.0405	0.3098	Genotyped
rs4791016	62317979	A	C	0.1445	0.9541	0.2974	Genotyped
rs17645023	62347495	A	T	0.2470	0.8583	**5.34E-05**	Genotyped
rs12451912	62396346	A	G	0.3096	0.9795	0.4353	Imputed
rs4791001	62399766	G	T	0.4726	0.9880	0.6894	Imputed
rs740559	62401711	A	G	0.4181	0.9978	0.9813	Imputed
rs3826345	62409946	C	G	0.3796	1.0378	0.2096	Imputed
rs8065024	62412552	A	G	0.3592	0.9621	0.2209	Imputed
rs9892619	62415330	C	T	0.1693	0.9388	0.0275	Imputed
rs7225652	62418843	A	G	0.3225	0.9721	0.3452	Imputed
rs4790998	62419168	C	T	0.3554	1.0405	0.2195	Genotyped
rs8064884	62419497	A	G	0.1691	1.0654	0.0276	Imputed
rs713572	62421523	C	T	0.3129	1.0157	0.4628	Imputed
rs8071936	62429465	G	T	0.2941	1.0556	0.1640	Genotyped
rs719935	62430681	A	C	0.2930	1.0554	0.1622	Imputed
rs719934	62430855	A	G	0.2930	1.0554	0.1622	Imputed
rs7217856	62434863	C	T	0.4428	0.9946	0.9378	Imputed
rs9892538	62436297	C	T	0.4421	0.9949	0.9453	Imputed
rs1019105	62439122	A	T	0.2647	0.9513	0.0265	Imputed
rs16960478	62440013	G	T	0.3248	1.0575	0.1017	Imputed
rs1507504	62441670	C	T	0.2897	0.9478	0.1567	Imputed
rs3785581	62443224	G	T	0.2707	0.9533	0.0260	Imputed
rs1051774	62459514	C	T	0.2284	0.9645	0.2404	Imputed
rs9892020	62460904	C	T	0.2417	0.9723	0.3550	Imputed

rs7219283	62463355	A	T	0.4642	0.9642	0.1229	Imputed
rs4790993	62465677	C	T	0.4538	0.9635	0.1285	Imputed
rs2885928	62465697	C	T	0.1102	0.9525	0.3445	Genotyped
rs11654578	62466030	A	C	0.2877	1.0476	0.1582	Imputed
rs2159210	62466422	A	G	0.2694	1.0301	0.3854	Genotyped
rs1024609	62467382	A	G	0.0168	1.0540	0.5336	Imputed
rs16960487	62471535	A	G	0.1145	0.9438	0.2669	Genotyped
rs3785579	62472963	C	G	0.1751	1.0584	0.1713	Genotyped
rs7210865	62474597	A	T	0.1378	0.9158	0.0377	Imputed
rs11651301	62475788	A	G	0.1806	1.0255	0.5309	Imputed
rs2363843	62476248	C	T	0.1203	1.0490	0.3431	Genotyped
rs16960501	62479099	A	G	0.0816	1.0897	0.0851	Imputed
rs11079678	62479111	C	T	0.4174	0.9435	0.0164	Imputed
rs1799938	62482766	A	G	0.0785	1.0980	0.0356	Imputed
rs2684	62483067	C	T	0.0838	1.0585	0.2283	Imputed
rs7212075	62490732	C	G	0.0813	1.0515	0.2728	Imputed
rs7209433	62495370	A	G	0.0790	0.9511	0.2642	Imputed
rs6504486	62496558	C	T	0.0790	1.0513	0.2644	Imputed
rs16960539	62497586	C	T	0.3869	0.9495	0.0182	Imputed
rs9902312	62500766	C	T	0.3869	0.9495	0.0182	Imputed
rs4790989	62506189	C	T	0.3937	1.0554	0.0139	Imputed
rs4790988	62506329	A	G	0.3878	1.0529	0.0184	Imputed
rs9303515	62507829	A	G	0.0813	0.9517	0.2552	Imputed
rs720059	62512579	C	T	0.0790	1.0513	0.2644	Imputed
rs9897354	62516070	C	T	0.3861	1.0534	0.0174	Imputed
rs7214803	62526143	G	T	0.0848	0.9518	0.2265	Imputed
rs3785575	62538504	C	T	0.3947	0.9492	0.0156	Imputed
rs9332408	62539449	C	T	0.0784	1.0510	0.2648	Imputed
rs6504487	62553814	A	C	0.0784	1.0510	0.2649	Imputed
rs973225	62565330	A	G	0.0619	1.0103	0.8212	Imputed
rs4790890	62570804	A	C	0.3877	1.0522	0.0184	Imputed
rs4790889	62570977	C	T	0.3877	0.9504	0.0184	Imputed
rs3952344	62571051	C	T	0.0790	0.9495	0.2344	Imputed
rs12450155	62571383	A	C	0.0790	0.9495	0.2344	Imputed
rs9903088	62573244	C	T	0.3877	0.9504	0.0184	Imputed
rs8066479	62574686	C	T	0.0790	0.9495	0.2344	Imputed
rs3213754	62576336	A	G	0.3877	1.0522	0.0184	Imputed
rs4791310	62581331	A	G	0.3943	0.9481	0.0136	Imputed
rs16960615	62583125	A	G	0.3933	0.9480	0.0136	Imputed
rs12942297	62622946	A	G	0.0783	0.9498	0.2334	Imputed
rs9303516	62630981	A	G	0.0784	1.0529	0.2326	Imputed
rs10512514	62642338	A	C	0.3876	1.0517	0.0182	Imputed
rs7211695	62666433	A	G	0.0807	0.9505	0.2246	Imputed
rs12603609	62669482	A	G	0.3873	0.9506	0.0177	Imputed
rs34310593	62682591	A	G	0.0605	0.9909	0.8365	Imputed

Supplemental Table S7. Detailed information of dataset used in meta-analysis.

Sample	Ancestry	Case (n)	Control (n)
International Schizophrenia Consortium (ISC-Aberdeen)	UK	720	698
International Schizophrenia Consortium (ISC-Cardiff)	Bulgaria	527	609
International Schizophrenia Consortium (ISC-Dublin)	Ireland	270	860
Interational Schizophrenia Consortium (ISC-Edinburgh)	UK	368	284
International Schizophrenia Consortium, Sweden 2 (ISC-SW2)	Sweden	390	229

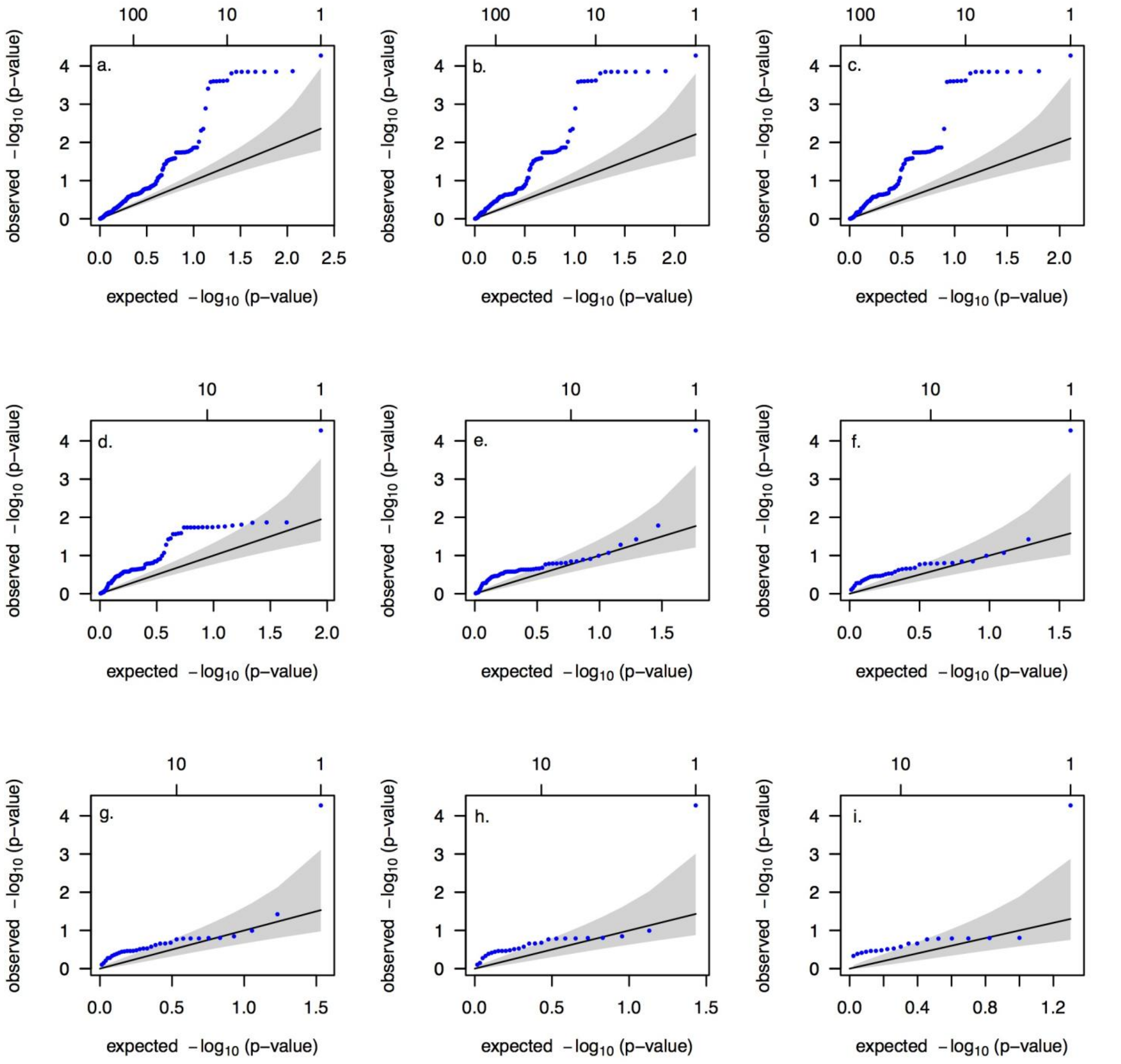

Supplemental Figure S1. Q-Q plots of the association *P* values for both imputed and genotyped SNPs using different info metric threshold. We chose the info metric threshold from 0.1-0.9 (a-i).
